# The Efficacy and Outcome of a Two-Staged Operation for Irreducible Knee Dislocation: A Prospective Short-Term Follow-Up

**DOI:** 10.3389/fbioe.2022.861788

**Published:** 2022-04-25

**Authors:** Shengyu Cui, Hong Yi, Xinhui Zhu, Jianbo Fan, Yi Ding, Wei Liu

**Affiliations:** ^1^ Department of Orthopedic Surgery, The Second Affiliated Hospital of Nantong University, Nantong, China; ^2^ Nantong First Peoples Hospital, Nantong, China; ^3^ Rehabilitation Hospital Affiliated to National Research Center for Rehabilitation Technical Aids, Beijing, China

**Keywords:** irreducible knee dislocation, knee stability, staged-surgery, medial collateral ligament, cruciate ligament, rehabilitation

## Abstract

**Background:** Irreducible knee dislocation (IKD) is a very rare but serious type of knee dislocation; it can lead to soft tissue necrosis due to incarceration of the medial structures and faces great difficulty in the postoperative rehabilitation, too. IKD needs careful pre-operative planning. There is no universal agreement about the appropriate surgical strategy for IKD. The purpose of this study was to investigate the clinical efficacy, safety, and outcome of the two-staged operation in treatment of IKD.

**Methods:** IKD patients were included from June 1, 2016 to May 31, 2020. In the stage-1 surgery, acute reduction and extra-articular structure repair were performed. Following an intermediate rehabilitation, delayed cruciate ligament reconstructions were performed in stage-2. Physical examination, CT, MRI, and X-ray were performed during the pre-operative period. Knee function, joint stability, ligament laxity, knee range of motion (ROM), and alignment were accessed at follow-ups. The minimum and maximum follow-up times were 0.5 years and 1 year, respectively.

**Results:** In total, 17 IKD patients were included. There were three subjects (17.65%) missing at the 1 year follow-up and the average follow-up was 11.18 ± 2.53 months. After stage-1, normal alignment and superior valgus/varus stability were restored in most subjects; however, a notable anterior–posterior instability still existed in most patients. The intermediate rehabilitation processed smoothly (6.94 ± 1.20 weeks), and all patients achieved knee ROM of 0–120° finally. At 0.5 years and 1 year follow-up after stage-2, all subjects had achieved normal knee stability, ROM, and satisfying joint function. No infection or DVT was observed.

**Conclusions:** The two-staged operation for IKD has superior efficacy on knee stability and function, and it can facilitate the rehabilitation and achieve satisfactory short-term outcome.

## Introduction

Irreducible knee dislocation (IKD) is a rare but serious type of knee injury (Malik and Macdonald, 2019), constituting up to 4% of all knee dislocation (KD) (Robertson et al., 2006; Schaefer et al., 2018). IKD is considered irreducible because of the soft tissue incarceration during closed reduction, for example, the capsuloretinacular structure, medial collateral ligament (MCL), vastus medialis muscle, or the gastrocnemius muscle are likely to trap in the medial compartment (Chen et al., 2011; Price et al., 2019). The “pucker sign” or “dimple sign” is considered as the diagnostic characteristic of IKD, which is due to invagination of the soft tissue into joint space (Jeevannavar and Shettar, 2013).

IKDs belong to the KD-IIIM (Solarino et al., 2015), according to Schenck’s (Jr, 1994) classification. IKD is a great challenge for orthopedists and needs very careful preoperative planning. First, IKD should be treated urgently in case of some severe complications, such as knee flexion contracture (KFC) and soft tissue necrosis (Hill and Rana, 1981). Second, multiple ligament injury always faces difficulties in rehabilitation. It has been reported that knee functional prognosis can be seriously compromised in IKD patients (Hill and Rana, 1981).

Recently, literature has favored the two-staged surgery with intermediate rehabilitation. The stage-1 surgery consists of acute reduction with extra-articular structure repair (Solarino et al., 2015), followed by an intermediate aggressive rehabilitation, and the stage-2 surgery consists of delayed ACL and PCL reconstruction (Levy et al., 2009; Howells et al., 2011; Burrus et al., 2016). Several case reports have indicated that the two-staged surgery combined with intermediate rehabilitation facilitated the recovery of ROM and knee function (Levy et al., 2009; Howells et al., 2011; Burrus et al., 2016) or resulted in a better outcome of the joint stability and function (Mook et al., 2009; Jang et al., 2014). However, as the IKD case is very limited, only several case reports exist (Malik and Macdonald, 2019), lacking systematic clinical and follow-up studies. At present, there is no universal agreement about the appropriate operating time or stage for treating IKD (Malik and Macdonald, 2019). The purpose of this study was to investigate the clinical efficacy, safety, and outcome of the two-staged operation in treatment of IKD.

## Materials and Methods

### Patient Involvement

**FIGURE 1:**
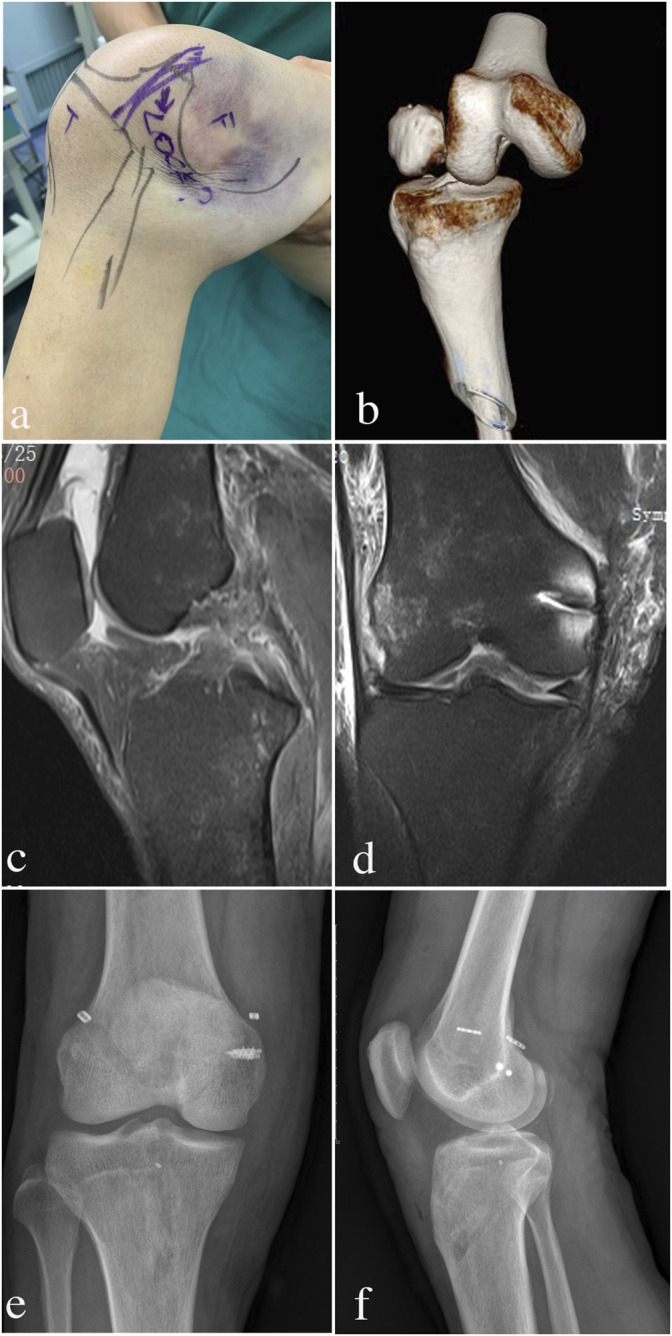
Evolution process of IKD patient appearance and imaging examinations from injury to postoperative follow-up. **(A)** “Dimple sign” and severe ecchymosis with invagination of skin and soft tissue can be observed on the medical side; **(B)** before the stage-1 operation, CT showed the knee posterolateral dislocation; **(C)**: before stage-2 operation, MRI showed that the MCL structure had been repaired; d: before stage-2 operation, MRI showed the ACL and PCL rupture; **(E,F)**: X-ray showed normal knee alignments at 0.5 years and 1 year follow-up.

IKD patients were included between June 1, 2016, and May 31, 2020, in our department. The inclusion criteria were as follows: 1) age: 18–60 years old, BMI≤31; 2) KD-IIIM (multiligament knee injury with ACL, PCL, and MCL ruptured) (Solarino et al., 2015; Jr, 1994) (Figures 1A, B); 3) irreducible (the closed reduction could not be performed); 4) the two-staged surgery was performed; 5) the stage-1 surgery was performed in 1 week post injury. The exclusion criteria were as follows: 1) KD-IIIL (multiligament knee injury with ACL, PCL, and LCL + PLC rupture) (Jr, 1994; Solarino et al., 2015); 2) history of lower extremity fracture, ligament rupture, and operations; 3) rheumatoid arthritis, ankylosing spondylitis, Parkinson’s disease, or other nervous system diseases.

The protocols and procedures for the protection of human subjects were approved by the Ethics Committee in our hospital (IRB ethical approval: KY-2016-OB08), and all of the methods were conducted in accordance with the approved guidelines.

### The Two-Staged Surgery and Intermediate Rehabilitation

In the beginning, vascular and nerve injury assessment was performed on the patients. The two-staged operation was performed by the same senior surgeon. Knee X-ray and CT were performed before the operation.

**FIGURE 2:**
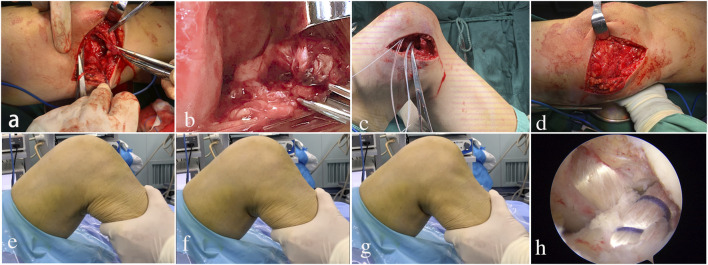
Two-staged operation procedures. **(A,B)**: in stage-1, the damaged MCL and other medial structures were exposed, which were found interposed into the condylar notch; **(C,D)**: the torn MCL and medial retinaculum were replaced and repaired, and the dislocated knee was back to normal alignment following the suture of the medial joint capsule; **(E–G)**: before stage-2 began, the drawer tests found notable anterior-posterior instability, that is, the anterior drawer test, **(F)**, neutral position, **(G)**, the posterior drawer test; **(H)**: the ACL and PCL reconstruction in stage-2.

The stage-1 operation consisted of urgent open reduction and extra-articular structure (MCL, medial retinaculum, and capsule) repair. With a pneumatic tourniquet inflated on the thigh, a 5-cm longitudinal incision was made proximally from the medial femoral epicondyle, distally to the lower border of pes anserinus. This reverted the skin and subcutaneous fat flap posteriorly and exposed the damaged medial structures (Figure 2A), which were incarcerated into the condylar notch (Figure 2B). The torn MCL, medial retinaculum, and capsule were replaced and meticulously sutured with interrupted #1 absorbable braided sutures. The ruptured MCL and medial gastrocnemius muscle from the bony attachment were reinserted at its origin with a suture anchor (Figure 2C). Finally, the dislocated joint restored normal alignment after stage-1 (Figure 2D).

The intermediate rehabilitation lasted for 6–8 weeks (Ng et al., 2020). An above-the-knee plaster was applied with the joint flexed to about 20°. The patients were asked to follow the instructions for non–weight bearing for 2 weeks. Then, partly weight bearing was allowed and a rehabilitation program was started to restore the joint range of motion (ROM) of 0–120° and muscle strength (3rd–6th week). Full weight bearing was allowed in the 6th–8th week. The MRI, clinical assessment, and self-administered questionnaire (Lysholm and International Knee Documentation Committee (IKDC) score (Ranger et al., 2011)) were assessed after the rehabilitation.

The stage-2 operation was begun when the rehabilitation was accomplished with an ROM of 0–120°. All patients underwent the arthroscopic reconstruction of ACL and PCL (Figure 2H). Unilateral semitendinous and gracilis tendons were used as auto-graft. The femoral side was fixed using EndoButton CL (Smith and Nephew, Memphis, TN, United States), and the tibial side was fixed using the poly-l-lactic acid biodegradable interference screw.

### Follow-Up

The follow-up was started when stage-2 surgery was completed. The end was re-dislocation/death/missing, whichever occurred first. The minimum follow-up was 0.5 years, and the maximum was 1 year. General parameters included age, gender, body mass index (BMI), occupation, trauma level, MCL injury type, time between injury and stage-1, time between stage-1 and stage-2, follow-up time, and complications. Knee X-ray photographs, clinical assessments, and self-administered questionnaires (Lysholm and International Knee Documentation Committee (IKDC) score (Ranger et al., 2011)) were performed at 0.5 years and 1 year follow-up. All of the subject data were checked and entered into the database by two researchers, and double entry was used to carry out quality control.

### IKD Clinical Assessments

The physical examination for assessing joint stability of IKD included the Lachman test, posterior drawer test, pivot shift, and varus and valgus stress test (Ranger et al., 2011).

Ligament laxity was measured using KT-1000: the forward shift is tested when the knee is flexed at 30°, and the backward shift is tested when the knee is flexed at 70°. According to IKDC standards, laxity is evaluated by comparing to the healthy side: a difference of less than 3 mm is normal, 3–5 mm is close to normal, 6–10 mm is abnormal, and more than 10 mm is severe.

The ROM was measured using a standardized goniometry technique. The knee flexion contracture (KFC) was assessed by passive physical examination. KFC is defined as the gap value of extension loss compared to the normal side, and more than 5° was considered KFC according to the Knee Society Score (KSS) system (Insall et al., 1989).

### Statistical Analysis

The sample size was pre-calculated by using the following formula: *n*= (*μ*_α_+ *μ*_β_)^2^ σ^2^/δ^2^, *a* was set at 0.05, β was set at 0.01, and the power was 90% (1-β). The comparisons of the count data were processed by the chi-square test or Fisher’s exact test, and continuous data were processed by independent sample t-tests and Levene’s variance homogeneity tests. The level of significance was set at 0.05. All of the statistical analyses were performed using SPSS 20.0.0 (SPSS Inc., 2009; Chicago, IL, United States).

## Results

### Basic Characteristics

The calculated sample size was 14 (power: 90%). Finally, there were 17 IKD patients included in this study (Table 1). Most of the IKD patients were injured by low-energy trauma (*n* = 14): three subjects suffered a sprain of knee when jogging or other exercising; four sprained the knee by themselves during farming; two sprained themselves by accidents during indoor daily life; five were caused by motor vehicle accident. The others were caused by high-energy traffic accident (*n* = 2) or fall (*n* = 1). All of the patients were transferred by ambulance to the Emergency department, and urgent reduction (stage-1 surgery) was performed in 0–3 days (Table 1). The knee was fixed in a slight flexion and valgus posture with severe ecchymosis on the medial side, and sometimes the “dimple sign” can be observed (Figure 1A). CT showed posterolateral knee dislocation (Figure 1B). None of the patients had vascular (both the posterior tibial artery and dorsalis pedis artery) or peripheral nerve (both the sensory and motor) injury.

**TABLE 1:** Basic characteristics and clinical conditions at baseline of the follow-up.

Baseline Indicators	Details
Enrolled subjects	17
Age	42.47 ± 8.90 (range: 29–60)
Sex (male/female)	11/6
BMI	25.89 ± 2.14 (range: 22.00–29.10)
Trauma	Self-injured: nine cases
	Low-velocity motor vehicle accident: five cases
	High-velocity traffic accident: two cases
	Fall: one case
Occupation	Farmer: eight
	Manual worker: five
	Office worker: two
	Retired: two
Time to stage-1* surgery (day)	0.76 ± 0.90 (range: 0–3)
Duration from stage-1 to 2 (week)	6.94 ± 1.20 (range: 6–10)
Type of MCL injury	Femoral attachment: 6
	Tibial attachment: 4
	Mid-substance: 6
	Peel-off: 1
Follow-up time (month)	11.18 ± 2.53 (range: 6–14)
Subject number in 0.5 years	17
Subject number in 1 year	14 (missed 3)
Total missing	3/17
Complications	Stage-1: stiffness (2/17)
	Stage-2: hemarthrosis (3/17), fever (3/17)
	0.5 years: KFC (5/17)
	1 year: KFC (3/14)

Note: * stage-1, surgery was performed together with the urgent reduction.

BMI (body mass index).

### The Two-Staged Surgery and Intermediate Rehabilitation

The stage-1 surgery was performed in 0.76 ± 0.90 days after injury (Table 1). Two patients had reversible joint stiffness after the stage-1 surgery (Table 1), and they were intervened by physics therapy with extended rehabilitation for 1–2 weeks until an ROM of 0–120° was restored. No infection or DVT was observed.

After the intermediate rehabilitation, all patients had obtained normal knee alignment (Figure 1D), ROM (0–120°), and valgus/varus stability in the pre-stage-2 period (Table 2). MRI showed ACL and PCL ruptures in all patients (Figure 1C). Lachman and posterior drawer test also found notable anterior–posterior instability in most patients (Figures 2E–G).

**TABLE 2:** Clinical assessments of knee stability before stage-2 operation and during the follow-ups.

Physical Examination/Measurement		A: pre-Stage-2* (*n* = 17)	B: 0.5 years (*n* = 17)	C: 1 year (*n* = 14)	*P*_*A-C*_	*P*_*A-B*_
					*χ* ^2^	*χ* ^2^
Lachman test	−	0	17	14	<0.001	<0.001
	±	1	0	0	*χ* ^2^ *= 34.000*	*χ* ^2^ *= 31.000*
	+	16	0	0	*υ* = 2	*υ* = 2
Posterior draw	−	0	17	14	<0.001	<0.001
	±	2	0	0	*χ* ^2^ *= 34.000*	*χ* ^2^ *= 31.000*
	+	15	0	0	*υ* = 2	*υ* = 2
Pivot shift	−	0	17	14	<0.001	<0.001
	±	4	0	0	*χ* ^2^ *= 34.000*	*χ* ^2^ *= 31.000*
	+	13	0	0	*υ* = 2	*υ* = 2
Valgus stress	−	13	16	14	0.146	0.052
	±	4	1	0	*χ* ^2^ *= 2.110*	*χ* ^2^ *= 3.782*
	+	0	0	0	*υ* = 1	*υ* = 1
Varus stress	−	15	16	13	0.545	0.665
	±	2	1	1	*χ* ^2^ *= 0.366*	*χ* ^2^ *= 0.188*
	+	0	0	0	*υ* = 1	*υ* = 1
30° forward shift	<3 mm	0	14	12	<0.001	<0.001
	3–5 mm	0	3	0	*χ* ^2^ *= 34.000*	*χ* ^2^ *= 31.000*
	5–10 mm	2	0	0	*υ* = 3	*υ* = 3
	>10 mm	15	0	2		
70° backward shift	<3 mm	0	15	11	<0.001	<0.001
	3–5 mm	0	2	3	*χ* ^2^ *= 34.000*	*χ* ^2^ *= 31.000*
	5–10 mm	1	0	0	*υ* = 3	*υ* = 2
	>10 mm	16	0	0		

Note: *pre-stage-2: just before the stage-2, operation began, when the stage-1 operation and rehabilitation of 6–8 weeks had been accomplished; the 30° forward shift and 70° backward shift were performed by KT-1000; there was no difference between 0.5 years and 1 year follow-up.

The duration from stage-1 to stage-2 surgery was 6.94 ± 1.20 weeks (Table 1). Three subjects had hemarthrosis (10–20 ml) with transiently absorptive fever (<38.0°C) (Table 1), but those implications disappeared spontaneously in 1–2 days. Finally, all patients underwent the arthroscopic reconstruction of ACL and PCL with unilateral semitendinous and gracilis tendons as auto-grafts (Figure 2H).

### Follow-Ups

The average follow-up time was 11.18 ± 2.53 months (Table 1). There were three subjects missing at 1 year follow-up, who cannot be contacted through phone call/e-mail or claimed that they cannot participate anymore, and the total missing rate was 17.65% (Table 1). There were five and three subjects who had mild KFC at 0.5 years and 1 year follow-up, respectively (Table 1).No infection or DVT was observed during the follow-ups.

Most of the subjects had achieved normal joint stability during follow-ups (Table 2). The positive rates of Lachman, posterior drawer, pivot shift test, and forward and backward shift decreased significantly at 0.5 years and 1 year follow-up than those at the pre-stage-2 period, and no difference was found between 0.5 years and 1 year follow-up (Table 2). The valgus/varus instability rate showed no statistical difference among the three periods (Table 2).

**FIGURE 3:**
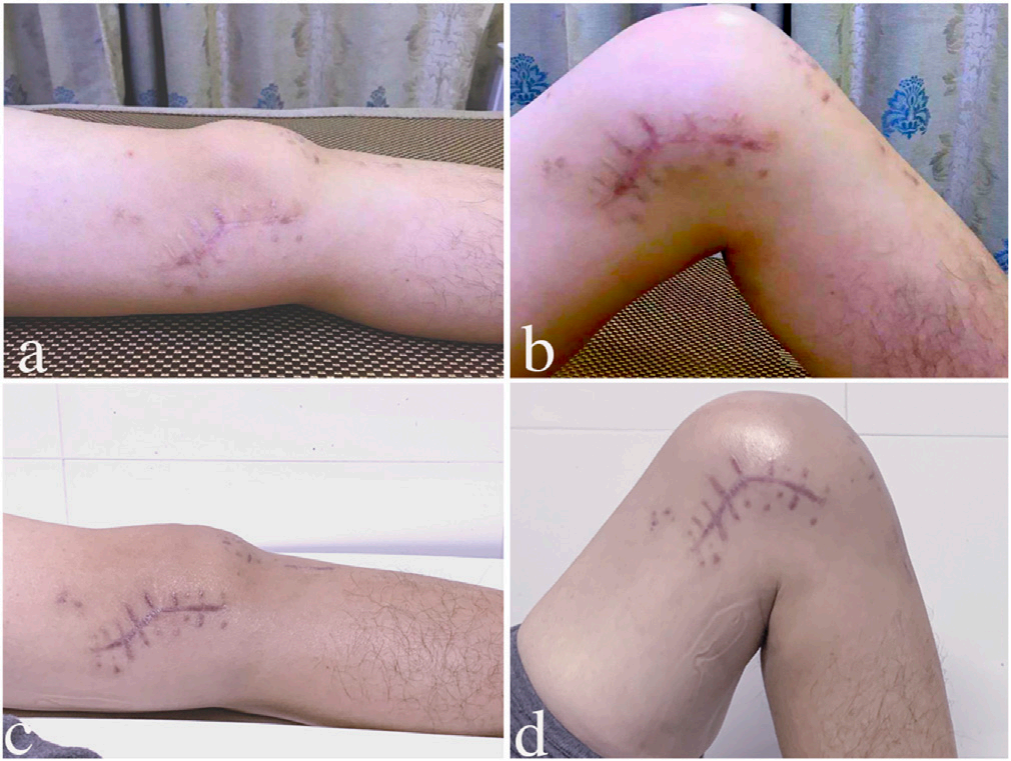
Knee ROM at the intermediate rehabilitation and 1-year follow-up. **(A,B)**: subject achieved a normal knee extension and flexion angle after the intermediate rehabilitation; **(C,D)**: at 1 year follow-up, the same patient’s knee extension and flexion angle showed a further increase.

Every subject had achieved a normal knee ROM at both follow-ups (Table 3). The knee extension angle at 0.5 years follow-up had an increasing trend compared to that in pre-stage-2, and there was no difference between 0.5 years and 1 year follow-up (Table 3). At 1 year follow-up, the knee extension angle showed a significant increase compared to that in pre-stage-2 (Table 3) (Figure 3). The knee flexion angle at 0.5 year follow-up showed a significant increase than that at pre-stage-2, and this angle was further increased at the 1 year follow-up (Table 3). The flexion angle at the 1 year follow-up was much bigger than that at pre-stage-2 (Table 3) (Figure 3). Lysholm and IKDC scores increased significantly at 0.5 years follow-up than those at pre-stage-2, and those parameters were further increased at the 1 year follow-up (Table 3). The normal knee alignment was observed at 0.5 years and 1 year follow-up (Figures 1E, F).

**TABLE 3:** Knee range of motion and joint function at the pre-stage-2 period and follow-ups.

Parameters	A: pre- B: 0.5 years Stage-2* (*n* = 17) (*n* = 17)	C: 1 year (*n* = 14)	*P*_*A-B*_ *t,υ*<	*P*_*A-C*_ *t,υ*	*P*_*B-C*_ *t,υ*
Knee extension −1.29 ± 1.26	−2.53 ± 2.27	−3.07 ± 2.46	0.058 *t = 1.963*	0.025 *t = 2.447*	0.529 *t = 0.637*
			*υ = 32*	*υ = 18.531*	*υ = 29*
Knee flexion 123.41 ± 4.08	128.88 ± 6.90	138.07 ± 8.89	0.008 *t = -2.814*	<0.001 *t = -5.697*	0.003 *t = -3.242*
			*υ = 32*	*υ = 17.472*	*υ = 29*
Lysholm 46.06 ± 6.43	77.12 ± 4.68	84.50 ± 4.50	<0.001 *t = -16.112*	<0.001 *t = -18.865*	<0.001 *t = -4.448*
			*υ = 32*	*υ = 29*	*υ = 29*
IKDC 11.22 ± 2.23	74.52 ± 3.06	79.00 ± 5.71	<0.001 *t = -16.112*	<0.001 *t = -14.402*	0.003 *t = -3.187*
			*υ = 32*	*υ = 29*	*υ = 29*

Note: *pre-stage-2: just before the stage-2, operation began, when the stage-1 operation and rehabilitation of 6–8 weeks had been accomplished; IKDC: International Knee Documentation Committee.

## Discussion

As IKD is a rare type of knee dislocation, most of the present studies were case reports or case series studies (Malik and Macdonald, 2019). In this study, the patients were enrolled from a province (local population: 84.8 million) in eastern China, where the climate is very rainy and locates on multiple interprovincial highways. The results found that most patients were farmers (8/17) and manual workers (5/17), and we considered that it can be attributed to the high risky environment characteristics for IKD. It has been reported that most IKDs are caused by low-energy trauma (Hussin et al., 2016; Hohmann et al., 2017; Schaefer et al., 2018), and the results showed that 82% IKD cases were caused by low-energy trauma (14/17), including sports traumas, self-spraining, and low-velocity motor vehicle accident.

IKD always needs very careful preoperative planning. Previous study has found that one-staged surgery may achieve high rates of arthrofibrosis and knee stiffness (Levy et al., 2009; Howells et al., 2011; Wu et al., 2014; Burrus et al., 2016) and postoperative instability and re-dislocation (Gu et al., 2004; Wu et al., 2014). It is supposed that the two-staged surgery combined with intermediate rehabilitation can obtain good efficacy and result in satisfying functional outcome (Levy et al., 2009; Mook et al., 2009; Howells et al., 2011; Jang et al., 2014; Burrus et al., 2016).

In this study, stage-1 surgery was performed within 3 weeks after injury because it is considered to be the critical time frame to repair the soft tissue without significant scarring (Ng et al., 2020). Our results showed that repairing of MCL and other extra-articular structures in stage-1 can provide sufficient anti-valgus stability, and it was maintained very well during the follow-ups. Soft tissue incarceration is the key feature of IKD: valgus stress separates the medial tibial plateau from the condyle, which increases the capsular volume and generates a negative pressure, trapping the medial structures into the condylar notch; as a result, the medial condyle dislocates the joint with soft tissue invagination (the ‘pucker sign’) (Jeevannavar and Shettar, 2013). Hence, the results indicated that urgent surgery is needed in order to minimize the risk of soft tissue necrosis. The MCL is the most powerful medial structure of the knee. Our results indicated that the MCL should be anatomically repaired in the stage-1 surgery of IKD as well in order to achieve superior healing capability (Kovachevich et al., 2009; Xu et al., 2017).

The intermediate aggressive rehabilitation facilitated the recovery of ROM and knee function. Once the normal ROM was restored, the stage-2 surgery (delayed reconstruction of ACL and PCL) was begun (Levy et al., 2009; Howells et al., 2011; Burrus et al., 2016). In this study, the stage-2 surgery of ACL and PCL reconstruction was performed based on normal ROM, knee alignment, and medial stability contributed by intermediate rehabilitation. We considered that both ACL and PCL reconstruction were essential for the IKD patients because of the results found.

A notable anterior/posterior instability and rotational instability existed commonly after the stage-1 surgery. Similar to our findings, Solarino et al. also reported that the IKD patients may experience a feeling of joint instability without ACL or PCL reconstruction even for elderly subjects (Solarino et al., 2015). However, Bistolfi et al. declaimed that cruciate ligament reconstruction can be avoided in elderly patients or subjects who were not professionally engaged in a high-level sport (Bistolfi et al., 2011). Our results found that a superior anterior–posterior stability and rotational stability can be achieved and maintained at least 1 year after the stage-2. It has been reported that the delayed cruciate ligament reconstruction (>3 weeks) offers better ROM and rehabilitation (Levy et al., 2009; Howells et al., 2011; Burrus et al., 2016). Our results indicate that both ACL and PCL should be reconstructed in the stage-2 surgery of IKD, contributing to joint stability and functional outcome.

On the other side, IKD always affects the pes anserinus tendon. The hamstring is one of the most commonly utilized auto-grafts for ligament reconstruction (Mahmoud et al., 2014), and the graft quality is of great significance in clinical efficacy and outcome (Moghamis et al., 2019). Hence, ligament reconstruction should be delayed in stage-2 (Solarino et al., 2015). The present study found that the intermediate rehabilitation (6–8 weeks) progressed very well after the stage-1, and only two cases of stiffness occurred, but they can be successfully reversed by physics therapy. Finally, all subjects started the stage-2 surgery with normal ROM and knee alignment. Our results suggest that the intermediate duration of 6–8 weeks is sufficient for the pes anserinus to restore, and unilateral auto-graft can provide sufficient tendon strength for both reconstructions.

Our results showed that all subjects had achieved a full ROM, superior joint stability, and continuously increased IKDC and Lysholm scores at follow-ups. Similarly, systematic reviews demonstrated that the two-staged surgery resulted in a good outcome on the joint stability and function in KD-III injuries, which was better than acute or delayed surgery (Mook et al., 2009; Jang et al., 2014). Our results indicated that the two-staged surgery with intermediate rehabilitation can have a satisfactory functional outcome.

Our study presents several limitations. First, as the incidence of IKD is very low, our sample size was small. Second, the follow-up time was not very long. A further longitudinal study with more samples and longer follow-up and different cohort studies are required to determine the long-term outcome of the two-staged operation.

## Conclusion

The two-staged surgery on treatment of IKD has superior efficacy on knee stability and function, and it can facilitate the rehabilitation and achieve a satisfactory short-term outcome.

## Data Availability

The data analyzed in this study is subject to the following licenses/restrictions: All of the data have been showed in this study. There are no additional unpublished data from this study. Requests to access these datasets should be directed to polocsy@163.com (SC).
